# Distribution and Characteristics of *Escherichia coli* Clonal Group A^1^

**DOI:** 10.3201/eid1101.040418

**Published:** 2005-01

**Authors:** James R. Johnson, Andrew C. Murray, Michael A. Kuskowski, Sören Schubert, Marie-Francoise Prère, Bertrand Picard, Raul Colodner, Raul Raz, and the Trans-Global Initiative for Antimicrobial Resistance Analysis (TIARA) Investigators

**Affiliations:** * VA Medical Center, Minneapolis, Minnesota, USA; † University of Minnesota, Minneapolis, Minnesota, USA; ‡ Max von Pettenkofer-Institut für Hygiene und Medizinische Mikrobiologie, München, Germany; § Centre Hospitalier Universitaire Purpan, Toulouse, France; ¶ Hôpital Morvan, Brest, France; # HaEmek Medical Center, Afula, Israel; ** Technion School of Medicine, Afula, Israel

**Keywords:** Escherichia coli, antimicrobial resistance, virulence, virulence factors, Escherichia coli infections, urinary tract infection, phylogeny, polymerase chain reaction, molecular epidemiology, dispatch

## Abstract

Among 1,102 recent *Escherichia coli* clinical isolates, clonal group A was identified in 17 of 20 (U.S. and non-U.S.) geographic locales, mainly among U.S. isolates (9% vs. 3%; p < 0.001) and those resistant to trimethoprim-sulfamethoxazole (10% vs. 1.7%; p < 0.001). The extensive antimicrobial resistance and virulence profiles of clonal group A may underlie its recent widespread emergence.

## The Study

The recently recognized *Escherichia coli* clonal group A (CGA) accounts for up to 50% of trimethoprim-sulfamethoxazole (TMP-SMZ)–resistant *E. coli* from U.S. women with acute uncomplicated cystitis and pyelonephritis (*1*–*3*). Available data show CGA to exhibit a stereotypical virulence factor (VF) profile and a conserved multidrug antimicrobial resistance phenotype, i.e., to ampicillin, chloramphenicol, streptomycin, sulfonamides, tetracycline, and trimethoprim (ACSSuTTp), which is conjugally transferable on a large plasmid (*1*,*2*). Together with CGA’s unusual O antigens (O11, O17, O73, and O77), these findings suggested that CGA represents a newly emerged virulent clonal group (*1*,*2*). CGA’s homogeneity across geographic locales, and the indistinguishable pulsed-field gel electrophoresis (PFGE) profiles of clustered CGA isolates within 1 community (*1*), suggested recent and possibly ongoing dissemination, a novel paradigm for extraintestinal pathogenic *E. coli* (ExPEC) (*4*).

To date, CGA has been studied only within the United States, predominantly among women with uncomplicated urinary tract infections (UTI). Its occurrence in some locales has been questioned (*5*), and its antimicrobial susceptibility profile has been assessed for only 12 drugs (*1*,*2*,*6*). Accordingly, we sought to more fully define the global distribution, host range, virulence characteristics, and resistance phenotypes of CGA.

Twenty clinical microbiology laboratories (10 U.S., 10 non-U.S.) each provided approximately 25 consecutive TMP-SMZ–resistant and TMP-SMZ–susceptible *E. coli* isolates, except for 2 that provided resistant isolates only (Table 1). Isolates were distributed by specimen type (urine/other) and source (inpatient/outpatient). Antimicrobial drug–resistant and –susceptible isolates were collected approximately concurrently in 2001 from each site, except London (1999) (*7*) and Columbus (1999 and 2002). When available, data regarding specimen type and host gender, age, and inpatient/outpatient status were provided.

**Table 1:** Sources of *Escherichia coli* clinical isolates and local prevalence of *E. coli* clonal group A (CGA) by trimethoprim-sulfamethoxazole (TMP-SMZ) phenotype*

Location	Type of institution	Patient population	Specimen type(s)	I/P or O/P†	Local TMP-SMZ resistance rate (%)	Total no. isolates	Prevalence of CGA, proportion (%)‡		p value
							TMP-SMZ–susceptible	TMP-SMZ–resistant	
Curitiba, Brazil	University medical center	Mostly adults	Urine	Unknown	33	60	1/30 (3)	2/30 (7)	
Montreal, Canada	University medical center	Mostly adults	Urine, other	I/P, O/P	33	50	0/25 (0)	0/25 (0)	
London, England	Teaching hospital	Mostly adults	Urine	Unknown	29	46	n.a.	0/46 (0)	
Brest, France	University medical center	Mostly adults	Urine, blood	I/P, O/P	30	59	0/29 (0)	0/30 (0)	
Toulouse, France	Children’s hospital	Children	Urine, other	I/P, O/P	24	50	0/25 (0)	5/25 (20)	0.05
Münich, Germany	University medical center	Mostly adults	Unknown	Unknown	24	55	0/27 (0)	2/28 (14)	
Afula, Israel	Outpatient clinic	Women	Urine	O/P	30	100	1/51 (2)	3/49 (6)	
Barcelona, Spain	Private medical center	Mostly adults	Urine, other	I/P, O/P	37	74	2/30 (7)	0/44 (0)	
Göteborg, Sweden	University medical center	Mostly adults	Unknown	Unknown	10%	48	0/25 (0)	1/23 (4)	
Bangkok, Thailand	Government hospital	Adults	Urine, other	I/P, O/P	67	48	0/24 (0)	1/24 (4)	
Baltimore, MD, USA	Student health center	Mostly women	Urine	O/P	29	62	2/24 (8)	7/38 (18)	
Billings, MT, USA	Private medical center	Mostly adults	Urine, other	I/P, O/P	11	50	0/25 (0)	3/25 (12)	
Birmingham, AL, USA	University medical center	Mostly adults	Urine, other	I/P, O/P	18	48	1/23 (4)	1/25 (4)	
Chicago, IL, USA	County medical center	Mostly adults	Urine	O/P	24	24	NA	7/24 (29)	
Columbus, OH, USA	University medical center	Children	Urine	I/P, O/P	13	59	0/33 (0)	6/26 (23)	0.005
Iowa City, IA, USA	(Multiple)	Unknown	Urine, other	Unknown	18	58	0/27 (0)	4/31 (13)	
Houston, TX, USA	Children’s medical center	Children	Urine, other	I/P, O/P	36	60	1/32 (3)	7/28 (25)	0.02
Rochester, MN, USA	Private medical center	Mostly adults	Urine, other	I/P, O/P	16	54	1/28 (4)	0/26 (0)	
Seattle, WA, USA	Teaching hospital	Adults	Unknown	Unknown	15	50	0/24 (0)	6/26 (23)	0.02
Tucson, AZ, USA	Veterans medical center	Men	Unknown	Unknown	25	47	0/34 (0)	2/13 (15)	

*Isolates were collected in 2001 for all sites except London, England (1999), and Columbus, Ohio (1999 for susceptible isolates and part of resistant isolates; 2002 for remaining resistant isolates). †I/P, inpatient; O/P, outpatient. ‡Eighteen centers contributed both susceptible and resistant isolates (total, 47 to 100 isolates each; median, 55). Two centers contributed only resistant isolates (Chicago, IL: 24 isolates; England: 46 isolates). Specimen type was known for 55 of the CGA isolates and was urine for 42, nonurine for 13 (10 blood, 1 tracheal aspirate, 1 fecal, 1 unspecified nonurine).

Phylogenetic group (A, B1, B2, or D) was defined by triplex polymerase chain reaction (PCR) (*8*). Group D isolates were defined as CGA if by random amplified polymorphic DNA (RAPD) analysis they resembled CGA controls with primers 1254 and 1290, or with one of these plus ≥2 of primers 1247, 1281, or 1283 (*2*). PFGE analysis used *Xba*I (*1*).

CGA isolates and (2:1) geographically matched controls selected randomly based on the local TMP-SMZ resistance prevalence were tested for 35 ExPEC-associated virulence markers and 13 *papA* alleles by PCR (*2*). Such typing predicts experimental in vivo virulence (*9*). Isolates were defined as ExPEC if positive for > 2 of *papA* and/or *papC*, *sfa/foc*, *afa/dra*, *iutA*, and *kpsM* (*10*). The aggregate virulence score was the number of different virulence markers detected in an isolate, adjusted for multiple detection of certain operons.

O typing was carried out by the *E. coli* Reference Center (University Park, PA). O antigens O1, O2, O4, O6, O7, O16, O18, O25, and O75 were considered UTI-associated (O-UTI) (*10*).

Susceptibility testing of the isolates to 30 drugs was carried out by broth microdilution (*11*) or disk diffusion (nitrofurantoin) (*11*), by using *E. coli* ATCC 25299 for reference and National Committee for Clinical Laboratory Standards (NCCLS)–specified interpretative criteria (*12*), except for kanamycin, spectinomycin, and streptomycin (resistant at MICs of >25 mg/L, >128 mg/L, and >8 mg/L, respectively). The aggregate resistance score was the number of different agents to which an isolate exhibited resistance.

Statistical analysis used a Fisher exact or a chi-square test for comparisons of proportions and the Mann-Whitney U test for comparisons of scores. Multiple variables were assessed as predictors of selected outcomes by multivariate logistic regression or multiple linear regression.

The 1,102 clinical *E. coli* isolates were divided evenly by U.S. and non-U.S. origin and TMP-SMZ resistance status (Table 2). Overall, phylogenetic group B2 predominated (44%), followed by group D (27%). Group D was significantly more prevalent among U.S. than non-U.S. isolates (34% vs. 21%: p < 0.001). TMP-SMZ–resistant isolates exhibited significant shifts from group B2 toward groups A, D, or both. The nearly 2-fold greater prevalence of group D among TMP-SMZ-resistant isolates reversed, for U.S. isolates, the “B2 > D” pattern of susceptible isolates.

**Table 2:** Prevalence of phylogenetic groups and *Escherichia coli* clonal group A (CGA) according to locale and resistance phenotype

*E. coli* group†	Prevalence of indicated *E. coli* group by locale and TMP-SMZ* phenotype, no. (column %)											
	Total (N = 1,102)				United States (N = 512)				Non–United States (N = 590)			
	Overall	Susceptible (n = 516)	Resistant (n = 586)	p value‡	Overall	Susceptible (n = 250)	Resistant (n = 262)	p value‡	Overall	Susceptible (n = 266)	Resistant (n = 324)	p value‡
ECOR A	168 (15)	65 (13)	103 (18)	0.02	63 (12)	28 (11)	35 (13)	NS	105 (18)	37 (14)	68 (21)	0.03
ECOR B1	143 (13)	70 (14)	73 (12)	NS	59 (12)	27 (11)	32 (12)	NS	84 (14)	43 (16)	41 (13)	NS
ECOR B2	483 (44)§	277 (54)§	210 (35)	<0.001	213 (42)	134 (54)§	79 (30)§	<0.001	270 (46)	143 (54)§	127 (39)§	<0.001
ECOR D	298 (27)§	98 (19)§	200 (34)	<0.001	176 (34)	60 (24)§ ¶	116 (44)§#	<0.001	122 (21)	38 (14)§ ¶	84 (26)§#	0.001
CGA	68 (6)	9 (1.7)	59 (10)	<0.001	48 (9)	5 (2)	43 (16)	<0.001	20 (3.4)	4 (1.5)	16 (5)	0.02

*ECOR, *E. coli* reference †TMP-SMZ, trimethoprim-sulfamethoxazole. ‡p values (by Fisher exact test) for comparison of susceptible and resistant isolates are shown when <0.05. NS, not significant. §For prevalence of group B2 (versus group D) within same column; p ≤ 0.01, McNemar’s test. ¶For prevalence of group D among U.S. (vs. non-U.S.) susceptible isolates; p = 0.005 (Fisher exact test). #For prevalence of group D among U.S. (vs. non-U.S.) resistant isolates; p < 0.001 (Fisher exact test).

By RAPD analysis, 23% of group D isolates (6% overall) represented CGA (Table 2). CGA was strongly associated with TMP-SMZ resistance (10% of resistant isolates, 1.7% of susceptible: p < 0.001) and a U.S. origin (9% vs. 3%: p < 0.001). CGA accounted for 15% of U.S. TMP-SMZ–resistant isolates and 5% of those from abroad (p < 0.001), but for a similarly low proportion of U.S. (2%) and non-U.S. (1.5%) susceptible isolates. According to multivariate logistic regression analysis, after accounting for TMP-SMZ resistance and U.S. origin, none of the other source variables (specimen type and host gender, age, and inpatient/outpatient status) was significantly associated with CGA status or appreciably altered the association of TMP-SMZ resistance or U.S. origin with CGA status (not shown).

**Figure 1:**
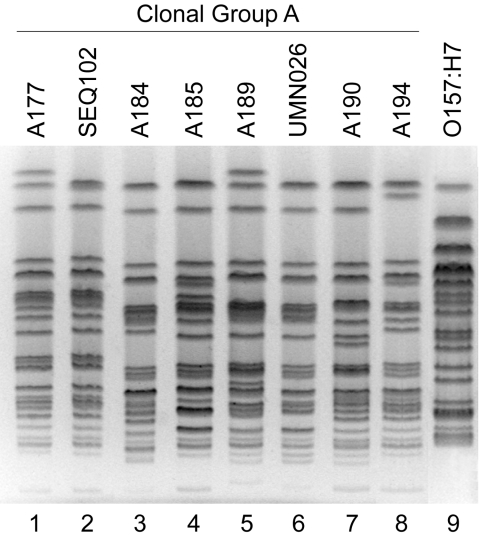
*Xba*I pulsed-field gel electrophoresis profiles of *Escherichia coli* clonal group A (CGA) isolates and *E. coli* O157:H7. Lane numbers are shown below the gel image. Six CGA isolates from Chicago, IL (“A” series identifiers; lanes 1, 3, 4, 5, 7, and 8) exhibit similar profiles to reference CGA isolates SEQ102 (from California; lane 2) and UMN26 (from Minnesota; lane 6) (*1*). *E. coli* O157:H7 isolate G5244 (lane 9) exhibits a distinctive profile.

CGA occurred in all but 3 locales (1–10 isolates per locale), accounting for up to 29% of local resistant isolates (median, 9.5%) (Table 1). CGA was highly prevalent (20%–25%) among resistant isolates from the 3 pediatric centers (Columbus, Houston, and Toulouse) and the veterans’ hospital (Tucson). Toulouse exhibited the highest non-U.S. CGA prevalence, whereas nearby Brest lacked CGA (Table 1). CGA accounted for 29% of the ostensibly nonclonal (*5*) TMP-SMZ–resistant isolates from Chicago. Consistent with this, *Xba*I PFGE profiles of 6 Chicago and 2 reference CGA isolates were highly similar (Figure 1).

**Figure 2:**
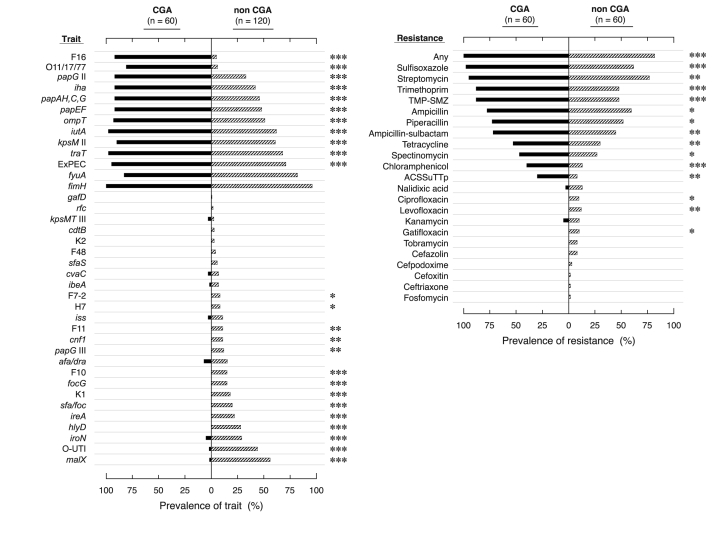
Virulence traits (top) and antimicrobial resistance phenotypes (bottom) of *Escherichia coli* clonal group A (CGA) isolates versus non-CGA controls. Percent of isolates positive for each virulence trait (top) or resistance phenotype (bottom) is shown by pink bars to left of midline (for CGA) or blue bars to right of midline (for controls). No isolates were positive for *bmaE* (M fimbriae), or resistant to amikacin, aztreonam, cefepime, ceftazidime, ertapenem, imipenem-cilistatin, nitrofurantoin, piperacillin-tazobactam, or ticarcillin-clavulanate. p value symbols at right of each chart, from Fisher exact test or χ^2^ test, are for significant differences between CGA and non-CGA. *, p < 0.05; **, p ≤ 0.01; ***, p ≤ 0.001.

CGA isolates differed significantly from controls, according to almost all characteristics analyzed; they were significantly enriched with the O11, O17, and O77 antigens, certain VFs (F16 *papA* allele, *papAHCEFG*, *papG* allele II, *iha*, *iutA*, *kpsM* II, *traT*, and *ompT*), and ExPEC status, but lacked other traits (Figure 2). Aggregate virulence scores were similar for CGA and controls (medians, 8.0) but were less diverse for CGA (range 5–9, vs. 1–13).

By multivariate logistic regression, both CGA status and phylogenetic group B2 were significant independent predictors of ExPEC status, with CGA exhibiting an odds ratio (OR) of 21.9 (95% confidence interval [CI], 6.1–78.2; p < 0.001), and group B2 an OR of 2.4 (CI, 1.7–3.4; p < 0.001). Additionally, by multiple linear regression, CGA status (standardized regression coefficient [β] = 0.57; p < 0.001) and group B2 status (β = 0.596; p < 0.001) predicted VF score better than did O-UTI (β = 0.32; p = 0.002).

Antimicrobial resistance phenotypes were compared between CGA isolates and a subset of the controls, which had been selected to provide an unbiased distribution of TMP-SMZ resistance (Figure 2). CGA exhibited a higher prevalence of resistance to 10 individual drugs, any drug, and ACSSuTTp, whereas controls exhibited a low but measurable prevalence of resistance to 7 drugs to which all CGA isolates were susceptible. Accordingly, resistance scores were significantly higher for CGA than controls (median 8.0 [range 1–11] vs. 5.5 [range 0–15]; p = 0.001). Among TMP-SMZ–resistant isolates, TMP-SMZ MICs ranged from 32/608 mg/L to >520/9,880 mg/L; 61% of resistant isolates had MICs >520/9,880 mg/L. CGA accounted for 67% of this highly resistant population (p = 0.01 vs. controls).

The U.S. and non-U.S. CGA isolates exhibited only 2 statistically significant differences for VF profiles, O antigens, and resistance phenotypes, with *iroN* (p = 0.03) and absence of *ompT* (p = 0.008) associated with non-U.S. isolates.

## Conclusions

We found that the recently described *E. coli* CGA is globally but heterogeneously distributed and more prevalent within the United States than abroad. Clonal group A affects diverse host populations (including inpatients, outpatients, adults, children, men, and women), infects urinary tract and nonurinary tract sites, is strongly associated with TMP-SMZ resistance (including high-level resistance), and exhibits a robust virulence profile suggesting enhanced extraintestinal virulence. This combination of resistance and virulence may account for CGA’s recent emergence as a broadly disseminated “epidemic clone” (*1*–*3*,*6*,*13*,*14*).

The greater overall prevalence of CGA within the United States suggests a U.S. origin for CGA. However, it also could reflect more rapid clonal expansion within the United States due to enhanced dissemination or more favorable conditions for outgrowth, including possibly less competition from other potential occupants of the same niche(s). The variable prevalence of CGA among even closely situated locales, both within the United States and abroad, might reflect true geographic heterogeneity, versus locale-specific differences in patient populations, selection criteria, and collection intervals. Studies that compare well-defined, homogeneous, concurrent populations from different locales are needed.

The new evidence of CGA as a prominent TMP-SMZ–resistant pathogen among children and veterans extends the known host range of CGA, consistent with a recent report of CGA as a community-wide TMP-SMZ-resistant pathogen in Denver, Colorado (*6*). This finding illustrates CGA’s pathogenic versatility.

That 29% of the TMP-SMZ–resistant isolates from Chicago, Illinois, proved to be CGA and resembled reference CGA isolates by PFGE, despite a previous PFGE-based assessment of nonclonality (*5*), illustrates the limitations of conventional PFGE analysis as a screen for CGA. We have developed a CGA-specific PCR assay, based on a single nucleotide polymorphism within *fumC*, to provide improved screening for CGA (*15*).

CGA’s robust, highly homogeneous consensus VF profile (F16 *papA* allele, *papG* allele II, *iutA*, *kpsM* II, *traT*, and *ompT*) suggests considerable extraintestinal virulence potential, an inference supported by experimental data indicating that CGA is able to compete successfully with classic group B2-derived pathogens in a mouse UTI model (J.R. Johnson, unpub. data). Determination of which VFs of CGA contribute most to pathogenicity may identify future targets for preventive interventions.

In our study, antimicrobial resistance was more extensive and, with TMP-SMZ, more potent for CGA than controls. These factors may be contributing to CGA’s global emergence. Conceivably, high-level TMP-SMZ resistance could allow CGA to out-compete even other TMP-SMZ–resistant strains. CGA’s resistance involved primarily older agents, whereas fluoroquinolone and cephalosporin resistance was confined to controls. However, the emergence of fluoroquinolone resistance within the closely related O15:K52:H1 clonal group (*2*,*13*) suggests that this potential exists also for CGA.

The study’s strengths include large population, broad geographic sampling, matching of CGA and control isolates, and extensive range of traits analyzed. Limitations include the heterogeneous inclusion criteria, gaps in global surveillance, possible type I errors from multiple comparisons, and limited sampling per locale.

In summary, we found CGA to be a globally disseminated, multidrug-resistant clonal group of pathogenic *E. coli* with a broad range of human hosts. Although more prevalent within the United States than abroad, CGA exhibits characteristic O antigens, resistance markers, and virulence traits wherever encountered. Further study is needed of the origins, virulence mechanisms, geographic distribution, clinical associations, and modes of dissemination of CGA.
